# Estimation in a multiplicative mixed model involving a genetic relationship matrix

**DOI:** 10.1186/1297-9686-41-33

**Published:** 2009-04-09

**Authors:** Alison M Kelly, Brian R Cullis, Arthur R Gilmour, John A Eccleston, Robin Thompson

**Affiliations:** 1 QDPI&F, Biometry, Toowoomba, Queensland, Australia; 2 NSWDPI, Biometrics, Wagga Wagga Agricultural Institute, Wagga Wagga, NSW, Australia; 3 NSWDPI, Biometrics, Orange Agricultural Institute, Orange, NSW, Australia; 4 School of Physical Sciences, University of Queensland, Brisbane, Queensland, Australia; 5 Rothamsted Research, Harpenden, Hertfordshire, AL5 2JQ, England, UK

## Abstract

Genetic models partitioning additive and non-additive genetic effects for populations tested in replicated multi-environment trials (METs) in a plant breeding program have recently been presented in the literature. For these data, the variance model involves the direct product of a large numerator relationship matrix **A**, and a complex structure for the genotype by environment interaction effects, generally of a factor analytic (FA) form. With MET data, we expect a high correlation in genotype rankings between environments, leading to non-positive definite covariance matrices. Estimation methods for reduced rank models have been derived for the FA formulation with independent genotypes, and we employ these estimation methods for the more complex case involving the numerator relationship matrix. We examine the performance of differing genetic models for MET data with an embedded pedigree structure, and consider the magnitude of the non-additive variance. The capacity of existing software packages to fit these complex models is largely due to the use of the sparse matrix methodology and the average information algorithm. Here, we present an extension to the standard formulation necessary for estimation with a factor analytic structure across multiple environments.

## Background

Selection of plants and animals in a breeding program deals with experimental data for which the underlying genetic model is best formulated as a mixed linear model. The genetic model is improved by including pedigree information through an additive relationship matrix, **A**. This matrix can be quite large and complex for large populations involving many generations, and its inverse is required when solving the mixed model equations. Efficient methods have been developed to permit routine application of this methodology. However, its application to multiple traits or environments in crop populations, where both additive and non-additive genetic variation can be measured, raises some issues to be resolved.

While pedigree information has been used extensively in animal breeding, adoption on a routine basis in the plant breeding sphere has been much slower. In cereal breeding programs, genotype performance is typically measured in a series of replicated field trials grown across multiple locations and years, and is collectively referred to as a multi-environment trial (MET), where current MET analyses assume independence between genotypes [1]. Benefits from the use of pedigree information can be two-fold. Firstly, the estimates of individual genotype performance are more accurate through the use of correlated information from relatives. In addition, breeding values can be estimated for each genotype, quantifying the potential of the individual as a parent in the breeding program.

One important aspect in the use of pedigree information in plant populations is the underlying genetic model, as additive and non-additive effects can be estimated separately [2]. This partitioning is possible since field crop data are generally from plots of genetically identical material, replicated both within and across environments. The additive component provides a simple covariance structure between related lines and the non-additive component is the lack of fit to the additive one. Crossa et al. [3] have fitted a genetic model including only an additive component, ignoring the non-additive variation. In our work, we investigate the performance of these different models and comment on the magnitude of the non-additive variation. The lack of fit can also be attributed to various forms of non-additivity including dominance [4] and additive by additive interaction [5] but we have not considered these more complex models.

The most general form for the genetic variance matrix from MET data is a fully unstructured matrix with *p *(*p *+ 1)/2 parameters where *p *is the number of environments, and this matrix is, by definition, nonnegative definite. For particular data, genotype effects are often highly correlated across some environments, leading to an estimated genetic covariance matrix that violates this condition; imposing constraints to force nonnegative definiteness leads to singular matrices, but standard REML methods require non-singular variance matrices. The magnitude of the estimation problem increases with the number of environments included, and the usual response is to replace the fully unstructured matrix with a more parsimonious approximation, the simplest of which has a common correlation across all environments. The factor analytic (FA) form introduced by Smith et al. [6] is intermediate in parsimony and is widely used in the analysis of MET data from most Australian plant breeding programs. Kelly et al. [7] have shown through simulation that this FA model is a robust model with high predictive accuracy. This model can accommodate increased correlation structure through incorporation of more factors, and can accommodate the singularity issue in the sparse matrix formulation presented by Thompson et al. [8].

When fitting a pedigree model across multiple environments, both Crossa et al. [3] and Oakey et al. [4] have adopted an FA model for the genotype by environment effects. Applications of the FA methodology have also recently arisen in the animal breeding literature, for example Meyer and Kirkpatrick [9] have fitted a constrained form of the factor model to animal pedigree data across multiple traits. However, problems arise in estimation methods for pedigree models combined with a complex variance structure across multiple environments or traits. Henderson [10] has presented a simple recursive method for computing the inverse of a relationship matrix, **A**^**-1**^, without the need to form the relationship matrix **A **itself. More recent improvements to the methodology have come from the work of Quaas [11] and Meuwissen and Luo [12], and this efficient algorithm is currently implemented in the software package ASReml [13]. For more complex variance models involving both the factor analytic and pedigree structure, the average information (AI) residual maximum likelihood (REML) methodology requires the formation of both elements of **A **and **A**^**-1 **^for the score equations and working variables. In this paper, we present the estimation approach used in ASReml for these more complex models and show how computational efficiency is maintained by only forming some elements of **A.**

In summary, this paper adopts the genetic model of Oakey et al. [2] with an extension to multi-environment trial data as the prototype [4]. We have investigated efficient model formulation and REML estimation of variance parameters for multiple environment/trait data using the standard approach in ASReml, with an extension for the factor analytic structure. An example of a multi-environment trial with pedigree structure is presented, and the goodness of fit of differing genetic models is considered.

## Methods

### A mixed model for MET data with pedigrees

Consider a series of *p *trials in which a total of *m *genotypes has been grown. Although *m *genotypes need not be tested in each trial, it is necessary to have adequate linkage between trials to estimate covariances. It is assumed that the *j*^*th *^trial comprises *n*_*j *_field plots and we let  be the total number of plots. A general mixed model for the *n *× 1 vector ***y ***of individual plot yields combined across trials can be written as

(1)

where **τ **is the *t *× 1 vector of fixed effects (typically environment means), **u**_g _is an *mp *× 1 vector of (random) genotype by environment effects, with associated design matrix, **Z**_g_, **u**_p _is a *b *× 1 vector of random effects (modelling design effects in the experiment), with corresponding design matrix, **Z**_p_, and **e **is the *n *× 1 vector of plot error effects combined across trials.

The random effects for genotypes can be partitioned according to the genetic model of Oakey et al. [2]. Additive effects can be estimated if pedigree information is available for the genotypes, and, if genotypes are replicated as they commonly are in METs, non-additive effects can also be estimated. The vector of genotype effects can be written as

(2)

where **u**_a _is the *mp *× 1 vector of (random) additive genotype effects and **u**_i _is the *mp *× 1 vector of (random) non-additive genotype effects, both ordered as genotypes within trials.

The random effects from equations (1) and (2) are assumed to follow a Gaussian distribution with zero mean and variance matrix

(3)

and

(4)

The variance matrix for the plot error effects is assumed to be block diagonal with **R **= diag (**R**_j_), where **R**_j _is the error variance matrix for the *j*^th ^trial. The variance matrix for extraneous random effects, **G**_p_, is usually a diagonal matrix of scaled identity matrices.

The partitioned genetic effects may each be represented as a two-way table of genotype by environment effects, and we assume that the variance matrix for the additive genotype effects has the separable form



where  and  are *p *× *p *and *m *× *m *symmetric positive definite matrices, respectively.  is the matrix of additive genetic variances and covariances between environments, and  is the variance/covariance matrix between genotypes. Following the approach of Oakey et al.[2], we set  = **A**, where **A **is a known numerator relationship matrix formed from pedigree information.

In a similar way, the non-additive effects may be represented as a two-way structure of genotype by environment effects, with an associated variance of



where  and  are also *p *× *p *and *m *× *m *symmetric positive definite matrices, respectively. We assume independence between the non-additive genotype components and hence set  = **I**_**m**_. The inclusion of this non-additive effect follows the model of Oakey *et al*. [2] and contrasts with the approach adopted by Crossa *et al*. [3] and Burgueno *et al*. [5], who choose to either omit the non-additive term, or model it as the interaction of additive effects,  = **A **# **A**, where # is the element-wise multiplication operator [5].

There are numerous possible choices for the form of  and . The form of the variance matrix adopted here is an FA model based on *k *factors, denoted FA*k*, and is given by



where  is a *p *× *k *matrix of environment loadings and **Ψ**_**a **_is a *p *× *p *diagonal matrix with elements commonly referred to as specific variances. In our model with partitioned genetic effects we will also be estimating parameters for the non-additive components, **Λ**_**i **_and **Ψ**_**i**_.

The particular form of the variance model for genetic effects to be estimated is,

(5)

and the plot variance from (4) and (5) is

(6)

Reduced rank models are a special case of the FA*k *model in which more than *k *of the specific variances are zero. The extreme of the reduced rank case is when all specific variances are constrained to be zero, as fitted in the fully reduced rank models proposed by Meyer and Kirkpatrick [9]. These models are denoted as FARR*k *models, for a *k*-dimensional FA model with all specific variances constrained to be zero.

Estimation of parameters in model (1) is achieved using two linked processes. Firstly, the variance parameters are estimated using REML [14]. This involves an iterative process, and in this paper the AI algorithm is used [15]. The second process involves estimation of Best Linear Unbiassed Predictors (BLUPs) of the random effects, and Best Linear Unbiassed Estimators (BLUEs) of the fixed effects in the model. As these effects are formed with estimated, rather than known, variance parameters they are referred to as empirical BLUEs and empirical BLUPs.

Thompson *et al*. [8] have described a method for estimation in reduced rank models with uncorrelated genotypes and we adapt this method for a relationship matrix, replacing **I**_m _with **A **and **A**^-1 ^as appropriate. A key issue for estimation with the more complex factor analytic models is that working variates require formation of **A**, in addition to **A**^**-1 **^as,

(7)

This requirement potentially reduces the efficiency of the methodology over simple pedigree models, which only require formation of **A**^-1^. To simplify the working variates, the standard approach in ASReml operates on the vector **ν =Aη**, obtained by directly solving the system of equations **A**^**-1 **^**ν = η**, using absorption and back substitution. This approach estimates only those elements of **A **that are required, and avoids having to completely form **A **as such, so that we can then substitute for **ν = Aη**, and proceed with routine application of the AI algorithm. We have considered an alternative formulation based on a Cholesky decomposition of **A**, but this introduced more dense matrices into the score and working variables. As such the formulation used in ASReml was the most efficient approach due to the sparsity of the **A **matrix, and the numerical methods used which capitalise on this property.

### Example Data set

The example data is a combined set of Stage 2 trials taken from the Queensland barley breeding program, grown in 2003 and 2004. Trial locations and dimensions together with mean yields for each trial are summarised in Table 1.

**Table 1:** Example barley data set: number of genotypes, trial dimensions and range in trial mean yield (t/ha)

Site	Year	Location	Number of genotypes	Trial dimensions		Mean yield (t/ha)
				Column	Row	
1	2003	Biloela	240	8	43	2.44
2	2003	Breeza	683	18	49	4.30
3	2003	Brookstead	460	8	74	1.25
4	2003	Clifton	460	8	74	1.42
5	2003	Kurumbul	685	8	111	1.74
6	2003	Narrabri	459	16	36	4.06
7	2003	Tamworth	456	8	72	3.89
8	2004	Billa Billa	719	8	110	1.91
9	2004	Biloela	172	8	28	4.58
10	2004	Breeza	720	20	44	4.00
11	2004	Brookstead	440	8	70	2.59
12	2004	Gilgandra	446	8	70	3.63
13	2004	Narrabri	455	8	70	3.97
14	2004	Walgett	454	8	70	2.64

The series follows two years of trials in the breeding program, where genotypes progress through stages of selection. A total of 1255 unique genotypes were tested in this series of trials, with 698 and 720 genotypes tested in 2003 and 2004, respectively. A common set of 163 genotypes was tested in both years, and the level of concurrence between all trials is shown in Table 2. The pedigrees of these genotypes were traced back four generations, in order to calculate elements of the numerator relationship matrix, **A**.

**Table 2:** Concurrence of genotypes across 14 barley trials

Site														
1	240													
2	237	683												
3	236	459	460											
4	236	460	238	460										
5	229	672	449	449	685									
6	235	457	382	310	450	459								
7	236	456	311	383	445	235	456							
8	15	163	93	85	158	91	86	719						
9	15	163	93	85	158	91	86	172	172					
10	15	163	93	85	158	91	86	719	172	720				
11	15	163	93	85	158	91	86	440	172	440	440			
12	15	162	92	85	157	90	86	446	171	446	270	446		
13	15	163	93	85	158	91	86	454	172	455	343	274	455	
14	15	163	93	85	158	91	86	454	172	454	343	183	354	454

Site	1	2	3	4	5	6	7	8	9	10	11	12	13	14

Total number of genotypes in each trial is on the diagonal of the table

Partially replicated designs [16], were used for all 14 trials in this series. Each dataset was analysed using the methods described in Section 2. A simple diagonal model for  and  failed to detect the presence of non-additive genetic variance at five of the 14 sites, so these were fixed to zero. In addition, four of the specific variances in the FA model for the nine sites were constrained to be zero, implying that the single latent factor explains all of the non-additive variance for these sites.

Four general classes of genetic model are examined. The first involves fitting the genotype effects as independent, fitting  but not  as a standard FA model [6]. The second class of model fits , but not , following the approach of Crossa *et al*. [3], who chose to omit non-additive genetic effects. The third class of model fits both components [4], in a model akin to Equation (5). Finally, fully reduced rank factor analytic models are considered, where the particular form of the variance structure for additive effects is constrained to follow the model of Meyer and Kirkpatrick [9].

The common element in all models for genetic variance is an FA structure for the genetic variance matrix. Each model begins with an FA structure of order 1, and progresses through higher dimensions as dictated by REML ratio tests (REMLRT). In the standard FA model, specific variances are constrained to be zero when they tend to estimates on the boundary of parameter space. In the fully reduced rank (FARR) model all specific variances are constrained to be zero, as this FARR structure deals solely with the factor component of the model.

To account for model parsimony, an Akaike Information criteria (AIC) is calculated for each model, and models are compared by forming the difference in AIC between each model and the best model.

## Results

The model with maximum REML log-likelihood and significant improvement in REMLRT over subsequent nested models is Model 8 in Table 3, which includes an FA structure of order 3 for additive effects, and an FA structure or order 1 for non-additive effects. It comes from a class of models proposed by Oakey *et al*. [4], in which Models 6 and 7 are lower order FA models, and involves fitting 71 genetic variance parameters through two FA structures. Model 8 is considered the best model based on the criterion of minimum AIC. The performance of other models is now considered in greater detail.

**Table 3:** Summary of REML logl-likelihoods and minimum Akaike Information Criterion (AIC) for the range of genetic variance models fitted to the example data set

Model	Structure of var(u_g_)		Number of		Log-likelihood	AIC¶
	**^†^**	**^‡^**	**parameters**	**Zero ****^§^**		

1	-	FA1	28	-	2366.4	1493
2	-	FA2	41	-	2477.3	1297
3	-	FA3	53	-	2504.7	1266
4	FA1	-	28	0	3051.1	124
5	FA2	-	41	0	3120.8	10
6	FA1	FA1 (9)	42	0	3115.1	24
7	FA2	FA1 (9)	55	1	3138.2	3
8	FA3	FA1 (9)	66	1	3150.9	0
9	FARR1	FA1 (9)	28	14	2525.3	1175
10	FARR2	FA1 (9)	41	14	2750.9	750
11	FARR3	FA1 (9)	53	14	2974.9	326
12	FARR4	FA1 (9)	64	14	3046.5	205
13	FARR5	FA1 (9)	74	14	3107.9	102
14	FARR6	FA1 (9)	83	14	3149.4	37

† genetic variance matrix for additive effects

‡ genetic variance matrix for non-additive effects

§ specific variances in the FA model for additive effects

¶ difference between each model and the best model

The simplest models for genetic variance, and those currently used in plant breeding programs in Australia, are Models 1–3, assuming independence between genotypes, (Table 3). Of these three models, the model of best fit is Model 3 with an FA structure of three dimensions. However it is inferior to all models incorporating pedigree information (Models 4–14).

The second class of model fitted, Models 4 and 5, involves only additive genetic variance, and does not capitalise on replication of genotypes and partitioning of non-additive effects. While these models are superior to those assuming independent genotypes, they are still inferior to the genetic model that partitions additive and non-additive effects, (Models 6–8).

The remaining models (Models 6–14) differ purely in the model for additive effects. The first subset (Models 6–8) involves an FA structure for  of increasing dimension, and the model with maximum likelihood is taken from this subset. The reduced rank (FARR) models impose a constraint on the more general FA model, and it can be noted that this constraint results in models of poorer fit for the same number of FA dimensions. In fact, six dimensions must be fitted in the reduced rank form (FARR6) to produce equivalent likelihoods to the best FA model with three dimensions. The AIC comparison also indicates that the general FA model produces a more parsimonious form than the FARR models.

In terms of the actual estimated parameters, we observed heterogeneity of both additive and non-additive genetic variance and heterogeneity of error variances across environments. Summaries of estimates of these variance parameters from the best model for  and  are given in Table 4. Genetic covariance in this data set was also heterogeneous and in our experience this is also typical of most multi-environment trial data. Genetic correlations were predominantly positive but there were instances where some pairs of trials had low/zero genetic correlation.

**Table 4:** Summary of parameter estimates from the best model for  and  for the example data set: genetic variance (diagonal elements of  and ) and error variance for each trial

Site	Year	Location	Additive variance	Non-additive variance	Error variance
1	2003	Biloela	0.1515	0.0154	0.1561
2	2003	Breeza	0.1281	-	0.1365
3	2003	Brookstead	0.0705	0.0256	0.0969
4	2003	Clifton	0.0138	0.0087	0.0411
5	2003	Kurumbul	0.0178	0.0097	0.3062
6	2003	Narrabri	0.1771	0.0932	0.1828
7	2003	Tamworth	0.1893	0.0561	0.1320
8	2004	Billa Billa	0.0875	0.0006	0.0323
9	2004	Biloela	0.1036	-	0.0847
10	2004	Breeza	0.9973	0.0283	0.1695
11	2004	Brookstead	0.1154	-	0.2608
12	2004	Gilgandra	0.2728	0.0172	0.0409
13	2004	Narrabri	0.1595	-	0.1159
14	2004	Walgett	0.1553	-	0.1350

**Figure 1:**
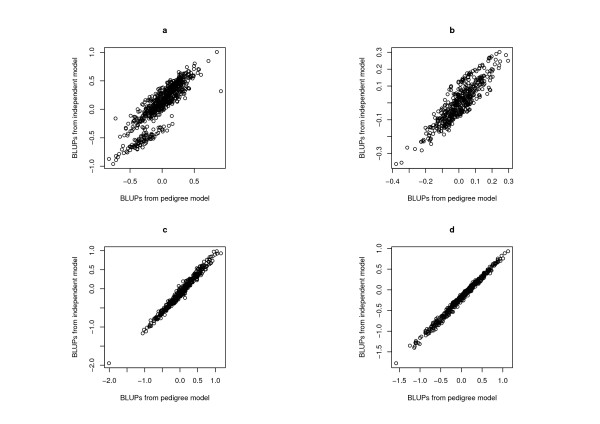
**Plot of predicted yield from two competing MET analysis models for four sites from the example data**. (a) 2003 Biloela, (b) 2003 Clifton, (c) 2003 Tamworth, (d) 2004 Gilgandra.

Of greatest importance to a breeding program is the impact of new analysis models on selection decisions. By examining changes in the empirical BLUPs between competing models, we can assess any changes in the ranking of the genotypes and subsequent changes in the selected subset of genotypes. Figure 1 displays the empirical BLUPs from the 'best' pedigree model, consisting of an FA3 model for additive effects combined with an FA1 model for non-additive effects, against the empirical BLUPs from the standard FA3 model assuming independence between genotypes. These plots are demonstrated using a subset of four sites. There is close agreement in rankings of empirical BLUPs for sites (c) and (d), where only six and two different genotypes are included in the top 46 genotypes (which forms the top 10%), respectively. These sites represent those with moderate and low levels of error variance, relative to additive genetic variance, (see Table 4). For sites (a) and (b) the empirical BLUPs deviate more from the one-to-one relationship, with 17 genotypes differing in the ranks of the top 46 genotypes.

The four sites in Figure 1 were chosen to demonstrate the different types of patterns evident in genotype predictions between the competing models. The relativity of additive genetic variance to error variance varies markedly between all sites, and while there is some consistency in genotype prediction for the sites with low error variance, there are many and varied patterns for sites with low to moderate levels of additive variance relative to error. Also, no consistent pattern in genotype predictions based on the relative magnitude of additive and non-additive variance is observed. For example, the site in Figure 1(a) has a very low proportion of non-additive variance estimated in the model, while plots (b) and (c) have the same proportion of non-additive variance (relative to total variance), with vastly different patterns between predictions.

It is also obvious from the banding patterns in Figure 1(a) and 1(b) that genotypes are regressing to a different underlying response in the pedigree model. The additive component provides a simple covariance structure between related lines and the non-additive component is the lack of fit to the additive one. Incorporation of the additive covariance in the model means each line is regressed toward the level predicted by its relatives, rather than to a common level for all genotypes, and reflects the theory of breeding by selection of parents for the next generation. The banding patterns in plots (a) and (b) result from the same cross, where the performance of individuals within this cross is elevated in plot (a) and depressed in plot (b). These differential predictions demonstrate the interaction between additive genetic variance and environment.

## Discussion

The inclusion of pedigree information in the analysis of MET data adds to the complexity of the mixed model and associated variance structure. Most plant breeding trials consist of replicated plot data across multiple environments, with an underlying variance structure for spatial effects and heterogeneity of variance at the residual level. Current analysis methods for MET data adopt a factor analytic variance structure for genetic correlation between environments. When the pedigree structure is added to model the relationship between genotypes, the resulting mixed model is quite complex, requiring the estimation of numerous variance parameters, and subsequent prediction of random genotype effects. The capacity of existing software to fit these complex models to 'real' data sets, (see ASReml) is largely due to the use of sparse matrix methodology and the AI algorithm [13].

In the analysis of the example data set, we investigate different genetic models for multi-environment data with a factor analytic variance structure. The genetic model of Oakey *et al*. [2], with an extension for MET data [4], adequately captures both the additive and non-additive genetic variation across environments, and is the model of best fit to the example data used in this study. Although only a small proportion of the total variation in the example data set is due to non-additive effects, a low order factor analytic model assuming independent genotypes still improved the goodness of fit. The genetic model with only additive effects [3], may be adequate when the level of non-additive genetic variance is low. Reduced rank models were less parsimonious than those with a standard FA form, requiring estimation of many more parameters from a greater number of dimensions to achieve an equivalent goodness of fit.

In theory, non-additive effects are comprised of the higher order interaction terms between additive and dominance effects [17]. In practice, the partitioning of the interaction variance is seldom more than trivial when compared with the errors of estimation [17]. While it is shown to be potentially beneficial to fit a simple model for non-additive variance, we surmise that partitioning into a complex model for non-additive effects [5] is unnecessary, as these often represent a relatively small proportion of the total genetic variance.

The improvement in model fit over the current model for MET data [6] is achieved through the inclusion of the numerator relationship matrix, **A**. In this paper, the relationship matrix is derived from pedigree information in the breeding program, but with the proliferation of molecular marker and quantitative trait loci data, elements of the genetic relationship matrix may now be derived in different ways [18]. For differing applications, the inter-individual relationships may be estimated, rather than assumed to be known, and methodology is available for estimating the elements of this correlation matrix, **A**. In these instances, it will not have the properties that allow **A**^**-1 **^to be an easily formed sparse matrix and this will limit the population size to which this empirical **A **matrix can be applied.

Of greatest importance to genetic gain in a breeding program is the impact of new analysis models on selection decisions. In this paper, we consider goodness of fit of each genetic model, and the impact of changes in rankings of empirical BLUPs of genotype effects between the pedigree and standard models. A large proportion of changes occur in the rankings of the genotypes at some environments, and we assume that the pedigree model would be predicting the most accurate effects. An additional benefit to selection of individuals and parents in the program is that the pedigree model estimates and adjusts for the interaction between additive genetic effects and environment.

An alternative way of assessing the impact on selection is through an improvement in prediction error variance (pev) of the empirical BLUPs from competing models. While for the pedigree model in our study the pev was reduced on average, we commonly overlook the fact that in this type of experiments, known biases are present in the pev and the empirical BLUPs themselves. The assumption of known **G **is violated as variance parameters must be estimated, and resulting empirical BLUPs and pev's are formed from , not **G**. Studies have shown that, while the properties of BLUPs do not hold under estimation of , the factor analytic models still perform well for empirical BLUPs [7]. A simulation study is required to examine the performance of empirical BLUPs for these more complex genetic models.

## Competing interests

The authors declare that they have no competing interests.

## Authors' contributions

AK completed the algebra to form mixed model estimates and validate software, and carried out the data analysis. RT and BC conceived the study, and BC and JE participated in its design and coordination. AG provided support for software and estimation methods. AK, AG, BC, and JE helped to draft the manuscript.
